# A randomised controlled trial of a family-group cognitive-behavioural (FGCB) preventive intervention for the children of parents with depression: short-term effects on symptoms and possible mechanisms

**DOI:** 10.1186/s13034-021-00394-2

**Published:** 2021-10-01

**Authors:** Johanna Löchner, Kornelija Starman-Wöhrle, Keisuke Takano, Lina Engelmann, Alessandra Voggt, Fabian Loy, Mirjam Bley, Dana Winogradow, Stephanie Hämmerle, Esther Neumeier, Inga Wermuth, Katharina Schmitt, Frans Oort, Gerd Schulte-Körne, Belinda Platt

**Affiliations:** 1 Department of Child and Adolescent Psychiatry, Psychosomatics and Psychotherapy, University Hospital, Ludwig-Maximilians-University, Munich, Germany; 2 German Youth Institute (Deutsches Jugendinstitut E.V.), Munich, Germany; 3 Department of Clinical Psychology and Psychotherapy, Ludwig-Maximilians-University, Munich, Germany; 4 Faculty of Social and Behavioral Sciences, Universiteit Van Amsterdam, Amsterdam, The Netherlands; 5 Institut für Therapieforschung, Munich, Germany

**Keywords:** Prevention of depression, Parental depression, Family intervention, High-risk

## Abstract

**Objective:**

Parental depression is one of the biggest risk factors for youth depression. This parallel randomized controlled trial evaluates the effectiveness of the German version of the family-group-cognitive-behavioral (FGCB) preventive intervention for children of depressed parents.

**Methods:**

Families with (i) a parent who has experienced depression and (ii) a healthy child aged 8–17 years (mean = 11.63; 53% female) were randomly allocated (blockwise; stratified by child age and parental depression) to the 12-session intervention (EG; *n* = 50) or no intervention (CG; usual care; n = 50). Self-reported (unblinded) outcomes were assessed immediately after the intervention (6 months). We hypothesized that CG children would show a greater increase in self-reported symptoms of depression (DIKJ) and internalising/externalising disorder (YSR/CBCL) over time compared to the EG. Intervention effects on secondary outcome variables emotion regulation (FEEL-KJ), attributional style (ASF-KJ), knowledge of depression and parenting style (ESI) were also expected. Study protocol (Belinda Platt, Pietsch, Krick, Oort, & Schulte-Körne, 2014) and trial registration (NCT02115880) reported elsewhere.

**Results:**

We found significant intervention effects on self-reported internalising ($\eta_{p}^{2}$ = 0.05) and externalising ($\eta_{p}^{2}$ = 0.08) symptoms but did not detect depressive symptoms or parent-reported psychopathology. Parental depression severity did not modify these effects. Both groups showed equally improved knowledge of depression ($\eta_{p}^{2}$ = 0.06). There were no intervention effects on emotion regulation, attributional style or parenting style.

**Conclusion:**

The German version of the FGCB intervention is effective in reducing symptoms of general psychopathology. There was no evidence that the mechanisms targeted in the intervention changed within the intervention period.

**Supplementary Information:**

The online version contains supplementary material available at 10.1186/s13034-021-00394-2.

## Introduction

### Parental depression as a risk factor

Depression is one of the most prevalent disorders and is predicted to be the world leading cause of illness by 2030 (WHO). One major risk for developing depression is having a parent who suffers from depression, which has been shown to be associated with adverse child outcomes, such as increased internalising and externalising symptoms [16, 20, 23, 24, 66, 67], and a more severe and continuous course of depression [24].

In the diathesis-stress model proposed by Goodman and Gotlib [23], four mechanisms explain how children of depressed parents are at greater risk of depression: (a) heritability, (b) innate dysfunctional neuro-regulatory factors, (c) exposure to negative maternal cognitions, behaviors, and affect, and (d) the stressful context of the children’s life. These are proposed to result in cognitive, affective and behavioural vulnerabilities in their offspring, which reduce the parental ability to cope with stress. Affective vulnerability maladaptive emotion regulation (ER) strategies (e.g. rumination, avoidance), which have consistently been shown to impact on the development of psychopathology [50] and may play a mediating role in the transgenerational transmission of maternal depression [54]. Moreover, adolescence goes along with using less adaptive but more maladaptive ER [15]. Cognitive vulnerabilities include a negative cognitive style (e.g. the tendency to draw negative interpretations from ambiguous situations), which may be transmitted from parent to child [17, 21, 28, 53]. A number of preventive interventions have been developed for the children of depressed parents and have demonstrated that it is possible to reduce children’s risk of depression (see [38] for a review and meta-analysis). Building on the model of Goodman and Gotlib [23], one key component of preventive interventions involves building resilience to stress by strengthening children’s cognitive, affective and emotional coping strategies, using techniques from cognitive-behavioural therapy (CBT) [9, 11]. A second component of preventive interventions is to improve family communication through psycho-education about depression [6, 11, 40]. By informing children about their parents’ depression, they might be capable of understanding the adverse emotional reaction and behavior by their parents. This may lead children to have an increased feeling of security and control [26, 37, 58]. A final component of preventive interventions is to provide parenting training. Depressive episodes have been shown to negatively impact parent–child interaction and strongly impair parenting [39, 62, 70]. By improving parenting consistency, preventive interventions may buffer against the effects of parental negative affect [11, 49].

Most existing preventive interventions for the children of depressed parents have focused on just one of the above components. For example, children’s coping strategies [9], communication within the family [6] or parenting training [49]. However, one intervention, the family group cognitive-behavioral (FGCB) preventive intervention for families of depressed parents [11], includes all three of the aforementioned elements: increasing children’s cognitive, affective and emotional coping strategies (children), family communication (family), and parenting training (parents). Compared to an active control group, the 12-session FGCB intervention was associated with reduced self-reported internalising symptoms immediately after the intervention (6 months from baseline) with a small effect size (ES) of *d* = 0.36 [14]. Effects on self-reported symptoms of depression and externalising symptoms were small and only emerged 12-months after baseline [14]. 24 months after baseline there was a very large positive effect of the intervention on the incidence of depression [14].

Furthermore, as is the case for most preventive interventions for the children of depressed parents, the FGCB intervention was developed in the United States (U.S.) and has not been evaluated elsewhere. Coping with mental illness can vary greatly between cultures. For example, cultures vary in the level of stigmatization of mental illness and the ease with which people feel able to share negative feelings with others [33, 45]. These differences in communication around mental health as well as parenting practices may impact the efficacy of group-based talking interventions such as the FGCB. A qualitative evaluation of the FGCB suggests that the intervention is accepted by families with depression in Germany [10]. However, it remains unknown to what extent the FGCB is effective in modifying children’s symptoms of psychopathology. Furthermore, it remains unknown whether the same factors which mediate and moderate the intervention in the U.S. also apply in Germany.

Since all existing interventions for the children of parents with depression show relatively modest effects, which diminish over time [38], understanding the degree of effectiveness of intervention components is essential for the development of improved interventions. In the original evaluation of the FGCB around half of the improvements in symptoms from 0 to 12-months for those in the intervention group were mediated by changes in children’s coping strategies and parents’ parenting style from 0 to 6 months [12]. Understanding which factors moderate the effects of interventions is important for the translation of interventions into routine practice. The most commonly studied moderator of interventions for children of depressed parents is the characteristics of parental depression when the family is enrolled in the intervention, e.g. whether the parent is depressed at baseline or not; how severely the parent suffers from depression. These findings are heterogeneous: in one large trial, children whose parents were not depressed at baseline benefited more from the intervention than those whose parents were experiencing a current depressive episode [6, 7, 22]. However, this finding could not be replicated in other trials [9, 11, 14]. A previous trial of the FGCB found moderating effects for a number of other variables, such as child age, child gender and parental education [14], although it should be noted that these effects were found for some but not all follow-up points. Another trial found that lower levels of functioning, higher levels of hopelessness and elevated symptoms of psychopathology of the children at baseline reduced the effects of an intervention (Weersing et al. 2016) although these findings are yet to be replicated.

In sum, a major limitation of the existing literature is the lack of information on which specific components of existing interventions work, and in which intervention settings.

### The current study

The first aim of the current study is to perform the first evaluation of the FGCB intervention outside of the original research group, in another country and culture. We seek to replicate previous findings [14] that the FGCB is associated with a lesser increase in self-reported internalising symptoms in children immediately after the intervention compared to a control condition. We also explore whether our adaptation of the intervention is effective in modifying self-reported externalising symptoms and depressive symptoms, as well as parent-reported psychopathology. Secondly, we investigate whether mechanisms that are targeted in the intervention (ER, attributional style, parenting and knowledge of depression) improve in the intervention versus the control group. Thirdly, we explore whether children whose parents are not depressed at baseline may benefit more from the intervention. Finally, since parental well-being is associated with their children’s symptomology [32], we investigate whether the intervention was associated with improvements in parents’ symptoms of depression, as it had been in the original study 12- [11] but not 15- [13] months after the intervention.

## Method

### Study design

**Fig. 1:**
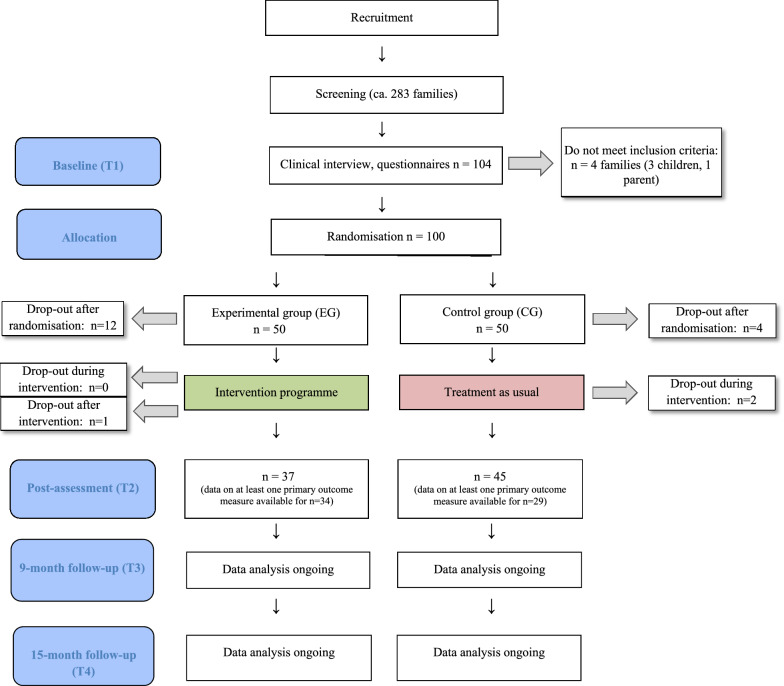
Study design

In a randomized controlled trial reported in line with the CONSORT statement (see Additional file 5), the FGCB intervention [11] was adapted to German (“Gesund und glücklich aufwachsen”; GuG-Auf) and evaluated. Psychiatrically healthy children and their parent(s) with depression were allocated either to the experimental group (EG) or to a usual care control group (CG). Figure 1 displays an overview of the study design. The randomisation procedure was performed by an independent researcher (FO), who was blinded to the identity of families. Randomisation was block-wise (per 10 recruited families^1^) and was stratified, based on whether parents were currently (versus remitted) depressed, as well as on the age of children. JL and KS-W enrolled participants and provided lists of family ID numbers and information necessary for stratification (parental depression status, child age). BP matched families based on stratification factors and provided blocks of family ID numbers to FO. FO generated the assignment sequence via computer and allocated families to groups. JL and KS-W were informed about which group each family had been assigned to by BP. All families were assessed at baseline (T1), immediately after the intervention (T2; 6 months) as well as nine- (T3) and fifteen-months (T4) after baseline.^2^ The sample size calculation was based on the primary outcome (episode of depression at T4) and assumed an effect size of *d* = 0.6, which was based on previous studies finding an incidence of depression in 33% of a no-intervention CG [5, 22] and 10% of an EG who received the FGCB [14]. In order to detect significant effects with an alpha level of 5% and power of 80% an a priori calculated one-sided Fisher’s exact test for determining the necessary sample size revealed a necessary *n* = 92 [47]. Although the sample was powered primarily to detect group differences in the onset of depression at T4, it assumed a medium ES of d = 0.6. This ES is in line with other studies of the effects of preventive interventions on symptom change post-intervention [14, 22].

### Participants

The 100 families (*EG* = 50, *CG* = 50) with 135 children were included in the study if a parent fulfilled the DSM-IV diagnostic criteria of a depressive disorder during the children’s lifetime and a child (aged 8–17, IQ > 85) did not meet the diagnostic criteria for a psychiatric disorder in the present or past. Additional siblings, who did not meet the study criteria (e.g. too young), were allowed to join intervention sessions provided they were not in crisis or did not suffer from severe psychological symptoms. Participants had to be fluent in German. Parents were excluded if they suffered from alcohol or substance abuse, bipolar disorder, reported psychotic symptoms, had a personality disorder or a suicidal crisis, in order to facilitate a workable group who predominantly share the experience of familial depression. Families taking part in family-based therapy that might interfere with the intervention effects were excluded. Each family received €25 at the beginning and the end of the study period to compensate them for their time. All participants were informed about the study procedure, possible risks and gave their written consent for study participation. The study was approved by the Ethics Committee of the Medical Faculty at Ludwig-Maximilian University (Study ID: 3–14). Families were recruited through public advertisements (32%), psychiatric hospitals and psychotherapists (26%), a city council database (17%), medical staff (12%), community centres (4%), previous studies (3%), word of mouth (3%), an information evening (2%) or unknown sources (1%).

### Study drop-outs

In total, 38 of 50 families randomised to the EG (76%) completed the intervention. The remaining twelve (24%) EG families dropped out after randomisation (but before the intervention began), largely due to time constraints (n = 8), or because the child had become affected by a psychiatric disorder herself (n = 1), although some families did not provide specific reasons (n = 2) or could not be contacted (n = 1). One family in the EG dropped out of the study after completing the intervention due to moving away from the area. Four of the 50 families randomised to the CG (8%) withdrew of the study after randomisation largely for unknown reasons (n = 3), but for one family because of marital disagreement about the risks of involving their children in a study about parental depression (n = 1). One additional CG family (2%) dropped out during the intervention period because the child no longer wanted to complete the questionnaires. Across both groups, post-intervention data were sometimes unavailable without families formally dropping out of the study (see "Missing data").

### Sample description

A description of the sample characteristics is provided in Table 1. 66 families lived in Munich and 34 in surrounding areas. Most of the sample comprised German nationals (91.5%) and a minority (8.5%) reported to have roots in Turkey, Croatia, Bulgaria and Austria. The families’ socio-economic status was high and parents were mostly well educated [56].^3^ Five parents in the EG and eight parents in the CG were single parents. There was evidence that the groups differed in positive parenting at baseline, indicating slightly more positive parenting skills in the EG (*t*_*1,99*_ = − 0.81, *p* = 0.039) and higher self-reported internalising symptoms in the children of the EG (*t*_*1,99*_ = 2.04, *p* = 0.044). Taking a closer look, the group difference in internalising symptoms was driven by one outlier in the EG (*M* = 35), since there were no group differences when this participant was excluded. The groups did not differ in any other variable (*ps* > 0.05).

**Table 1:** Demographic and clinical characteristics, children and parents at baseline

	EG	CG	Total	*p*-value
Children	n = 50	n = 50	N = 100	
Age, mean (SD)	11.73 (2.79)	12.04 (2.89)	11.89 (2.83)	0.596
Gender (%) female	55.1	52.0	53.5	0.760
IQ, mean (SD)	103.81 (14.21)	109.08 (13.18)	106.5 (13.88)	0.060
Siblings (%)	77.8	72.7	75.3	0.958
School type (%)^a^				
Primary school	31.0	34.1	32.5	0.822
Hauptschule	4.8	2.4	3.8	–
Realschule	14.3	9.8	12.0	–
Gymnasium	47.6	51.2	49.4	0.839

Parents	n = 50	n = 50	N = 100	
Age, mean (SD)	45.15 (5.80)	47.10 (7.01)	46.06 (6.43)	0.157
Gender (%) female	60.0	62.7	61.4	0.684
Highest level of education (%)				0.143
High school	14.0	18.2	15.8	
A-levels	23.3	30.3	26.3	
University	46.5	51.5	48.7	
Doctoral degree	16.3	0	9.2	
Family income (%)				0.704
– 2000 € /month	10.3	12.5	11.3	
2000–3000 €/month	17.9	18.8	18.3	
3000–4000 €/month	15.4	18.8	16.9	
4000–5000 €/month	30.8	25.0	28.2	
> 5000 €/month	25.6	25.0	25.4	
Depressive symptoms (BDI-II)	16.7 (10.04)	17.7 (12.29)	17.20 (11.10)	0.620
Currently depressed (%)	58.0	56.9	57.4	0.421
Treatment experience (%)				
Psychotherapy	92.3	94.3	93.2	0.504
Psychopharmaceuticals	82.1	69.7	76.4	0.165

^a^German secondary schools are categorised into three levels based on achievement at the end of primary school (increasing with grades): Hauptschule, Realschule or Gymnasium

*BDI-II *Beck’s depression inventory

### Psychopathology parents^4^

Most parents were diagnosed with a recurrent depressive disorder of mild (64.5%) or moderate (12.5%) severity, and 23% were in remission. Ten percent fulfilled the criteria for a double depression (experiencing episodes of major depression in addition to dysthymia). Only 14.8% had experienced a single depressive episode in their lifetime. 38% had comorbid diagnosis (mostly anxiety or eating disorders), 15% had slightly increased values on the personality disorder screening questionnaire (SKID II), but none showed clinically significant symptoms.^5^ 11.5% of the families consisted of two parents suffering from depression. The partner of parents with depression, who reported not to be affected by a mental illness, was also screened for psychopathological impairment using the SCL-90-R indicating 11% with current symptoms of a psychiatric disorder.

### Procedure

Families were recruited between July 2014 and October 2017. Follow-up data (T2) were collected between August 2015 and May 2018. Following a telephone-based screening, families were invited to the Department of Child and Adolescent Psychiatry at Ludwig-Maximilian University to receive more detailed information about the study, provide consent and take part in clinical interviews (with parents and children participating separately). Parents were given questionnaires to take home and to return within a week. Once 10 families were recruited, randomisation procedure took place and the families were informed about which group they were allocated to. At the end of the intervention (6 months after baseline), families received questionnaires and were asked to send them back.

## Measures

Table 2 gives an overview of the instruments used to determine eligibility for the study and outcome measures.

**Table 2:** Instruments used to measure eligibility criteria and outcome variables

Measure	Instrument
***Eligibility criteria***	
Diagnostic status (child)	K-DIPS psychiatric interview
Intelligence (child)	CFT 20-R test
Diagnostic status (parent)	DIPS psychiatric interview
Personality disorder (parent)	SKID II questionnaire
Psychopathology (2nd parent)	SCL-90-R questionnaire
***Main outcome variables***	
Depressive symptoms (child)	DIKJ questionnaire
Internalising and externalising symptoms (self- and parent-report)	YSR, CBCL questionnaire
***Secondary outcome***	
ER (child)	FEEL-KJ questionnaire
***Variables***	
Attributional style (child)	ASF questionnaire
Parenting style (child)	ESI questionnaire
Knowledge of depression (child)	Questionnaire
***Moderator variables***	
Depressive symptoms (parent)	BDI-II questionnaire

*ASF* Attributionsstil-Fragebogen für Kinder und Jugendliche, *BDI-II* Beck’s Depression Inventory, *CASE* Child and Adolescent Survey of Experiences, child and parent version, *CBCL* Child Behaviour Checklist, *CFT 20-R* Culture Fair Test 20. Revision, *DIKJ* Depressions-Inventar für Kinder und Jugendliche, *DIPS* Diagnostisches Interview bei psychischen Störungen, *ER* Emotion Regulation, *ESI* Erziehungsstil-Inventar, *FEEL-KJ* Fragebogen zur Erhebung der Emotionsregulation bei Kindern und Jugendlichen, *K-DIPS* Diagnostisches Interview bei psychischen Störungen im Kindes- und Jugendalter, *SCL-90-R* Symptom Checklist, *SKID II* Strukturiertes Klinisches Interview für DSM-IV, *YSR* Youth Self-Report

### Eligibility criteria

#### Diagnostic status of parents and children

The semi-structured clinical interview Diagnostisches Interview für Psychische Störungen [51] was administered to determine the diagnostic status of parents according to DSM-IV. Interrater reliability is high (*κ* = 0.72 and *κ* = 0.92) for general factors [61]. The child version, which includes interviews with parents and child, was used to confirm that no child met the criteria for any mental illness [63]. In cases where parents’ and children’s reports differed, the child report was given greater weight since parents suffering from depression tend to underestimate the impact of a child’s potential psychopathology symptoms [3]. To assess reliability in the current sample, twenty families were randomly selected and recordings of their interviews were rated independently by LT or KM. The pre-defined criterion was whether the person was currently depressed or not. The accordance rate for parents (n = 15)^6^ and children (*n* = 21) was excellent (100%; *κ* = 1.00).

#### Intelligence screening (child)

The Culture Fair Test (CFT 20-R, [65]) is an established multiple-choice intelligence assessment with five response options characterized by a very good re-test reliability (*r* = 0.96). It correlates well with other intelligence tests [49]. The CFT-20-R is split into two parts, with four sub tests each (serial continuation series, object classification, matrix and topologies). In this study, the short-version (part one; 56 items) was used.

### Outcome measures

#### Symptoms of depression (child)

The German version (DIKJ, [60] of the self-report Children’s Depression Inventory (CDI, [34]) contains 26-items scored on a 3-point Likert scale. The DIKJ has high internal consistency (Cronbach’s alpha *α* = 0.92, in this sample *α* = 0.86) and good temporal stability (re-test reliability *rtt* = 0.76). Construct validity is high, given that the items are directly based on the DSM-criteria for depression, external validity is moderate (positive correlations with self-esteem *r* = 0.60 and anxiety *r* = 0.61) [60]. In addition to the DIKJ, the depression and anxiety scale of the Child Behavior Checklist (CBCL, [19] and Youth Self-Report YSR, [18, 19]) were used to measure depressive symptoms.

#### Children’s internalising and externalising symptoms

German versions of the parent-report Child Behavior Checklist (CBCL, [18, 19]) and its self-report equivalent the Youth Self-Report (YSR, [18, 19]) were used. They contain 123-items with 3- or 4-point Likert scales (0 = “not at all” to 2 = “exactly true”). Internal consistency for the internalising and externalising subscales of the CBCL (*r* > 0.85) and YSR (*r* ≥ 0.86) is high. Here, internals consistency was lower (CBCL internalising *α* = 0.68, externalising *α* = 0.60; YSR internalising *α* = 0.79, externalising *α* = 0.73).

### Secondary outcome variables

#### Children’s knowledge of depression

The “Wissensfragebogen Depression” [1] assesses children’s knowledge of the symptoms and treatment of depression using 50 items (e.g. “suffering from depression means that someone is crazy” or “Depression means to be sad for a long period of time”) that are answered in a 4-point Likert scale (0 = “not at all true”, = “completely true”). The questionnaire has not yet been validated. The internal consistency was *α* = 0.64 in this sample.

#### Children’s ER

The self-report questionnaire *Fragebogen* *zur* *Erhebung* *der* *Emotionsregulation* *bei* *Kindern* *und* *Jugendlichen* (FEEL-KJ; [25]) assesses how children cope with the emotions anxiety, sadness and anger. It consists of 90 items that assess the use of adaptive (problem focused action, distraction, increased happiness, acceptance, forgetting, cognitive reappraisal, problem solving) and maladaptive (giving up, aggressive behavior, withdrawal, negative self-evaluation, perseveration) ER strategies. Each item (e.g. “When I’m angry, I keep my feelings to myself”) is rated on a 5-point Likert scale according to how often it is applied (“never” to “almost always”). The internal consistency was good for the adaptive (*α* = 0.95) and moderate for maladaptive (*α* = 0.82) in this sample. ER subscales (“Fragebogen zur Erhebung der Emotionsregulation bei Kindern und Jugendlichen—FEEL-KJ.,” n.d.) The six-week re-test-reliability for the two subscales is good (*r*_*tt*_ = 0.81, adaptive strategies; *r*_*tt*_ = 0.73, maladaptive strategies) (“Fragebogen zur Erhebung der Emotionsregulation bei Kindern und Jugendlichen—FEEL-KJ.,” n.d.). Sum scores for each emotion (clustering anger, anxiety and sadness).

#### Child attributional style

The Attributionsstil-Fragebogen für Kinder und Jugendliche (ASF-KJ; [59]) consists of eight positive and eight negative situations whose causes are rated on a 4-point scale in three dimensions: external vs. internal (1 = “caused by other person or circumstances” to 4 = “I caused this event”), instable vs. stable (1 = ”will never be important” to 4 = ”will always be very important”), and specific vs. global (1 = “is just this time relevant” to 4 = “will also be relevant at other occasions”), resulting in a total of six subscales. Reliability (Cronbach’s alpha) of the global and stability dimension lies between *α* = 0.72 and *α* = 0.81 and the internality dimension between *α* = 0.52 and *α* = 0.57 [59]. In this sample, we calculated the internal consistency of the positive subscale *α* = 0.87 and the negative subscale *α* = 0.82. Retest-Reliability (4 weeks) was acceptable (*r*_*tt*_ = 0.49 and *r*_*tt*_ = 0.65) [59]. We used sum scores of the three dimensions of positive and negative attributional scales.^7^

#### Parenting style

The parenting style inventory (ESI, [35]) is a 65-item child-report questionnaire covering positive (support, praise) and negative (criticism, restraint, inconsistency) parenting styles. Items are rated on a 4-point Likert scale (1 = “never or rarely happens” to 4 = “always happens”). The retest-reliability is modest (0.51–0.72), while the internal consistency of both, the mother’s and the father’s part, is good (0.77–0.92 and 0.65–0.71) [35]. Sum scores for positive (praise, support) (*α* = 0.92) and negative parenting (criticism, restraint, inconsistency) styles (*α* = 0.91) were calculated.

#### Parents’ symptoms of depression

To test whether parents’ symptoms of depression moderated the effects of the intervention, we used the German version of the 21-item Beck’s Depression Inventory (BDI-II, [27]). The BDI-II has a 4-point Likert scale (0 = “I do not feel sad” to 3 = “I am so sad or unhappy that I can’t stand it.”). Test re-test (5 months) reliability is r = 0.78 [27], Cronbach’s alpha was high in this sample with *α* = 0.96. Correlations with other measures of depression e.g. FDD-DSM-IV (Fragebogen zur Depressionsdiagnostik nach DSM IV, [28]) are high (*r* = 0.72–0.89).

#### Acceptance of the intervention

Participants completed anonymous feedback questionnaires at the end of each session (Additional file 2). Parents and children were asked to rate on a 5-point Likert scale (1) whether they understood the content of the session (1 = “not at all”; 5 = “very well”), (2) whether they participated actively (= “not at all”; 5 = “a lot”), (3) whether they felt comfortable, (4) whether they felt supported and understood by the group leader, (5) how well they understood the homework assignment and (6) how helpful the session was (3)-(6):1 = “not at all”; 5 = “very much”).

### Intervention

GuG-Auf (“Gesund und glücklich aufwachsen”) is the German adaptation of the FGCB [11]. The manualized intervention (available upon request) is a group-, family- and CBT-based intervention, combining parent–child and individual (parent and child only) sessions. The intervention lasts six months and includes eight weekly sessions, as well as four monthly booster sessions (120 min each) designed to trouble-shoot problems, which families may have when trying to apply the learnt skills in everyday life (see Additional file 1 for an overview of sessions). The intervention contains three basic components: psycho-education of parental depression (parents and children), stress coping strategies for children’s acceptance, distraction, positive thinking and positive activities^8^) and parenting training for parents (parenting and depression, displaying warmth and structure). Children and parents have homework tasks to complete between sessions, and parents are encouraged to spend at least 15 min of quality time a week with their children.

The FGCB manual was translated into German by a bilingual member of the research team (LBW). Ambiguities were discussed with the authors of the original intervention. Some cultural adaptations were made to the manual, including addressing parents using the formal “Sie” (children were addressed using the informal “du”), modifying leisure activities (e.g. football instead of baseball) and food examples (e.g. pretzels instead of crisps). In addition, more subtle cultural adaptations were made as considered appropriate for the German cultural context.^9^

Group leaders consisted of psychology doctoral students (KS-W, JL), trainee clinical psychologists (JL; LE, ED, KS, MB), or trainee child and adolescent psychiatrists (AV, IW, FL, SH). Each group was run by two leaders, with the majority of the sessions were conducted by JL (36%), KS-W (35%), LE (27%), AV (21%) and FL (20%). Additional trained members of the team jumped in when the main group leaders were unavailable: MB (12%), SH (9%), ED (9%), IW (8%), KS (8%). Occasionally, undergraduate psychology students ran the groups when those routinely conducting them were on sick leave: DW (12%), MK (5%), NC (3%). All group leaders received training in the intervention by BP, JL and KS-W and regular supervision (every session) by the principal investigators (BP, GSK).

Eleven groups of 3–5 families were run between January 2015 and June 2018. The sessions took place in the Department of Child and Adolescent Psychiatry of the Ludwig Maximilian University Hospital.

#### Fidelity of intervention

To enhance treatment fidelity, group leaders strictly followed the GuG-Auf manual. Upon initiation of the trial the original study team [11] provided the current research team with the manual and all associated contents. Multiple (online) training sessions took place between the original study team (Bruce Compas, Emily Hardcastle) and the core research team in Munich (BP, JL, KS-W). These sessions included role-plays and group discussions designed to communicate the core values of the intervention and identify and solve potential ambiguities. The remaining group leaders were trained by BP, JL und KS-W using the same techniques, In addition, the original research team answered questions and solved difficulties as they arose in the duration of the trial.

To evaluate treatment fidelity, two independent assessors (AH, LD) viewed a sub-sample of sessions and completed an adherence checklist (available upon request). Of the 220 videotaped sessions (sessions four to 11 contained separate videos for child- and parent-sessions), 55 (25%) were randomly selected, re-watched, and rated adherence. Ten of the 55 sessions had incomplete recordings of the full session (e.g. camera turned on too late), so that these parts of the session were not included in analysis. The average rate of completeness of intervention characteristics was high: 98.0% of sessions were fully completed (range 87–100%).

Attendance and homework completion lists were kept for all groups but data were missing for group 11. On average, families randomised to the intervention attended 7 sessions (range: 3–12). Of those families who attended at least one session, the average number of sessions attended was 9 and homework was completed by 69.79% of children and 60.63% of parents.

### Control condition

Unlike in the original trial [11], in which the control group received a brief written information sheet about depression, participants in the current control group received no active intervention. The no intervention condition, also adopted in a similar trial by Garber and colleagues [22] was chosen for clinical relevance i.e., to estimate the impact of the intervention above and beyond existing provisions. Indeed, it has been acknowledged elsewhere that whilst active-control conditions increase the internal validity of RCTs, they prohibit conclusions about the true preventive effect of an intervention [11]. Families in both conditions anecdotally reported receiving support from their GP or advice centres although whether this varied between the EG and CG was not systematically assessed. For ethical reasons, families in the CG were offered the intervention in some form after the study period.

### Analysis strategy

The data was analyzed using SPSS Version 19 (SPSS Inc., 1989–2006) for Windows and JASP Version 0.8.1.1. for Mac OX. An intention to treat approach was taken. T-tests on between-group (*EG,* *CG*) differences in the various outcome and confounding variables at baseline to check whether randomisation was successful.

Confounding variables, such as child age and gender, were intended to be included in analysis models. However, since neither variable was associated with changes in the outcome variables (all *ps* > 0.05) we ran repeated-measures ANOVA tests on each of the outcome variables,^10^ including the oldest eligible child in the family since risk of depression increases across adolescence. This also allowed us to test main effects of time in order to explore whether the intervention had modified the mechanisms it proposed to target, similar models were also run for the variables knowledge of depression, attributional style, parenting style and ER. The intervention effect on parental depression was analysed in the same way as the main outcomes. In addition, we tested the moderating effect on the outcome variables by including the dichotomous covariate of parental depression at baseline (being currently depressed yes/no) that was assessed in the clinical interviews with each parent. In families where both parents were suffering from depression, both were entitled to participate in the intervention but data from just one parent were included in the analyses.

Acceptance of the intervention was descriptively analysed by calculating mean scores from the feedback questionnaires, which were completed after each session (Additional file 3). Semi-structured interviews were conducted with a sub-set of families after the intervention and are reported elsewhere [10].

### Missing data

The range of missing outcome values varied from 10% (BDI-II T1) to 58.0% (DIKJ T2; Additional file 5). Importantly, the EG and CG groups did not differ significantly in the amount of missing data at baseline (t_1,99_ = 0.06; *p* = 0.415) or post-assessment (t_1,99_ = 0.17; *p* = 0.730). Thus, we assumed MAR [2] but due to the amount of missing data in the whole sample not MCAR. For the main analysis, missing values were imputed based on the expectation–maximization method [57].

## Results

Figure 1 provides an overview of the participant flow within the trial.

### Intervention effects on symptom severity

Table 3 provides means and standard deviations for each of the symptom severity variables (DIKJ, CBCL, YSR) before and after the intervention. Since the outcomes of the repeated-measures ANOVA test of group differences are reported in Table 3 (Additional file 2), they are not repeated in the text below.

**Table 3:** Statistical comparison of outcome variables (raw means, SD) at baseline and post-assessment

	Baseline		Post-assessment		Intervention effects (group x time effect)			
	EG	CG	EG	CG	F	df	*p*	$\eta_{p}^{2}$
Main outcome variables								
Depressive symptoms	8.24 (5.60)	7.49 (3.91)	8.28 (6.45)	5.67 (4.05)	2.49	1, 98	0.118	0.02
Internalising symptoms (self-report)	9.99 (7.34)	7.96 (5.34)	7.95 (6.90)	8.02 (5.24)	5.42	1, 98	**0.022**	0.05
Externalising symptoms (self-report)	9.03 (5.29)	8.73 (5.76)	7.82 (5.84)	10.42 (4.97)	8.44	1, 98	**0.005**	0.08
Depression/anxiety (self-report)	4.98 (3.18)	3.51 (2.56)	3.03 (3.97)	4.43 (3.12)	8.54	1,98	**0.004**	0.08
Internalising symptoms (parent-report)	9.01 (6.15)	8.97 (5.45)	7.22 (7.51)	8.14 (5.49)	0.76	1, 98	0.384	0.001
Externalising symptoms (parent-report)	7.17 (5.35)	6.34 (5.61)	5.59 (5.43)	4.41 (3.68)	0.88	1, 98	0.767	0.001
Depression/anxiety (parent-report)	4.48 (3.60)	4.06 (3.14)	3.46 (3.92)	3.71 (2.83)	0.96	1, 98	0.328	0.001
Potential mechanisms								
Adaptive ER	128.46 (26.91)	133.73 (26.37)	122.71 (35.34)	136.23 (24.45)	1.89	1, 98	0.172	0.002
Maladaptive ER	71.22 (13.02)	68.34 (14.21)	70.61 (14.15)	70.14 (11.08)	0.76	1, 98	0.383	0.001
Positive attributional style	66.06 (9.27)	65.66 (9.67)	69.21 (8.52)	70.03 (6.98)	0.51	1, 98	0.477	0.005
Negative attributional style	59.66 (9.49)	58.78 (10.83)	65.94 (8.57)	63.69 (8.08)	0.54	1, 98	0.465	0.005
Positive parenting	75.00 (9.48)	69.57 (10.21)	74.22 (12.68)	71.22 (11.40)	0.96	1, 98	0.330	0.010
Negative parenting	67.19 (12.52)	67.05 (9.63)	63.67 (10.41)	66.63 (7.52)	1.86	1, 98	0.175	0.019
Knowledge of depression	33.36 (4.26)	32.03 (2.99)	35.79 (2.40)	34.74 (2.98)	0.14	1, 98	0.704	0.001
Potential moderator								
Parental depression	16.70 (9.84)	17.76 (11.25)	11.96 (8.98)	13.92 (7.99)	0.17	1, 98	0.677	0.002

*ASF *Attributionsstil-Fragebogen für Kinder und Jugendliche, *BDI-II* Beck’s Depression Inventory, *CBCL *Child Behaviour Checklist, *DIKJ* Depressions-Inventar für Kinder und Jugendliche, *ER* Emotion Regulation, *ESI* Erziehungsstil-Inventar, *FEEL-KJ* Fragebogen zur Erhebung der Emotionsregulation bei Kindern und Jugendlichen, *YSR* Youth Self-Report

#### Self-reported symptoms of depression (DIKJ, YSR, CBCL)

Although both groups showed significant reductions in depressive symptoms over time (*F*_*1,98*_ = 5.78, *p* = 0.018, $\eta_{p}^{2}$ = 0.06), there were no significant differences between groups (*F*_*1,98*_ = 2.49, *p* = 0.118, $\eta_{p}^{2}$ = 0.02). Of note, we analysed anxiety-depression sub-scale of the YSR and found a significant intervention effect (*F*_*1,98*_ = 8.58, *p* = 0.004, $\eta_{p}^{2}$ $\eta_{p}^{2}$ = 0.08). Children in the EG reported significantly less symptoms over time (*m* = 1.94, *SD* = 5.69, *t* = 2.41, *p* = 0.019), while children in the CG showed more symptoms over time (*m* = − 1.02, *SD* = 4.00, *t* = − 1.77, *p* = 0.083). Parents in both groups reported significantly less anxiety-depressive symptoms over time (*F*_*1,98*_ = 3.97, *p* = 0.049, $\eta_{p}^{2}$ = 0.04), but there were no group differences in change over time. These results were not modified by parental depression at baseline (all *p*’s > 0.05).

#### Self-reported internalising and externalising symptoms

There was a significant difference between groups (*EG* vs. *CG*) on changes in self-rated internalising (*F*_*1,98*_ = 5.42, *p* = 0.022, $\eta_{p}^{2}$ = 0.05) and externalising symptoms from T1 to T2. The EG showed significantly fewer internalising symptoms from T1 to T2 (*m* = 1.95, *SD* = 6.13, *t* = 2.25, *p* = 0.029) whereas the CG did not change over time (*p* > 0.05). The EG showed no significant change in externalising symptoms over time (*p* > 0.05), whereas the CG showed an increase (*m* = − 1.69, *SD* = 5.19 t = − 2.31, *p* = 0.025). These results were not modified by parental depression at baseline (all *p*s > 0.05).

#### Parent-reported child internalising and externalising symptoms

There were no significant differences between groups (*EG,* *CG*) in changes in parent-reported internalising or externalising symptoms from T1 to T2. These results were not modified by parental depression at baseline (all *p*s > 0.05).

### Effects of potential mechanisms

#### Emotion regulation (ER)

There were no significant differences between groups (*EG,* *CG*) in changes in adaptive or maladaptive ER. These results were not modified by parental depression at baseline (all *p*’s > 0.05).

#### Attributional style

Both the EG (*m* = − 3.15, *SD* = 9.12, *t* = − 2.44, *p* = 0.018) and CG (*m* = − 4.37, *SD* = 7.87, *t* = − 3.92, *p* < 0.001) showed significantly more positive attributions over time (*F*_*1,98*_ = 19.47, *p* = 0.000, $\eta_{p}^{2}$= 0.166), but there was no difference between the groups. In addition, both the EG (*m* = − 6.27, *SD* = 10.42*,* *t* = − 4.26, *p* < 0.001) and CG (*m* = − 4.92, *SD* = 7.87, *t* = − 4.42, *p* < 0.001) showed increased negative attributional thinking (*F*_*1,98*_ = 36.76, *p* = 0.000, $\eta_{p}^{2}$ = 0.273), but there was no difference between the groups (*F*_*1,98*_ = 0.54, *p* = 0.465, $\eta_{p}^{2}$ = 0.005). These results were not modified by parental depression at baseline (all *p*s > 0.05).

#### Parenting Style

There was no evidence of group differences in changes in positive (*F*_*1,98*_ = 0.96, *p* = 0.330, $\eta_{p}^{2}$ = 0.001) or negative (F_1,98_ = 1.860, *p* = 0.175, $\eta_{p}^{2}$ = 0.019) parenting style. These results were not modified by parental depression at baseline (all *p*s > 0.05).

#### Knowledge of depression

Although both groups showed more knowledge of depression over time (*F*_*1,98*_ = 45.97, *p* < 0.000, $\eta_{p}^{2}$ = 0.32; *EG*: *m* = − 2.42, *SD* = 4.06, *t* = − 4.21, *p* < 0.001; *CG*: *m* = -2.71, *SD* = 3.47, *t* = − 5.51, *p* < 0.001), there was no evidence that this differed between the groups (*F*_*1,98*_ = 0.14, *p* = 0.704, $\eta_{p}^{2}$ = 0.001) and was modified by parental depression at baseline (all *p*s > 0.05).

### Parental depression

Parents in both groups showed a significant reduction in depressive symptoms from T1 to T2 (*F*_*1,98*_ = 15.75, *p* < 0.001, $\eta_{p}^{2}$ = 0.138), but did not differ from each other (*F*_*1,98*_ = 0.17, *p* = 0.677, $\eta_{p}^{2}$ = 0.002).

### Acceptance of intervention

Descriptive ratings of the intervention on six scales according to parents and children are provided in the Additional files 3 and 4. Averaging across all sessions, parents provided a mean rating of 4.35 of a possible 5 (*SD* = 0.71) and children’s ratings were similar (*M* = 4.35, *SD* = 0.83).

## Discussion

This RCT aimed to evaluate the effectiveness of GuG-Auf: the German adaptation of the family- and group-based cognitive behavioural (FGCB) intervention for the children of depressed parents [11]. The original intervention showed positive effects in modifying self-reported internalising symptoms immediately after the intervention [14]. This study is the first to attempt to replicate these effects outside of the USA and in an independent research group.

### Summary of findings

As expected, there was a significant effect of the intervention on children’s self-reported internalising symptoms with a medium to large ES: children in the CG showed no change over time whereas children who received the intervention showed a decrease over time. Similar findings (this time a large ES) were also observed for children’s self-reported externalising symptoms: this time children in the CG showed an increase over time whereas children in the EG showed no change. Contrary to expectations, there was no effect of the intervention on children’s self-reported depressive symptoms or parent-rated child psychopathology. The intervention had no effects on the proposed target mechanisms: although the EG showed significant (and very large) improvements in their knowledge of depression, so did the CG. Parental depression severity reduced in both groups over time with a large effect. Baseline parental depression severity did not have an impact on any of the outcome variables. The acceptance of the intervention was good.

### Effects of the intervention on symptom severity

The finding that children in the EG showed improved internalising symptoms whereas children in the CG showed a worsening of symptoms over time is in line with other studies of preventive interventions for children of depressed parents [6, 9, 14, 22, 38, 49]. A meta-analysis of preventive interventions for children of depressed parents found a small ES for internalising symptoms [38]. The ES in the current study is small-to-medium ($\eta_{p}^{2}$ = 0.05 is equivalent to *d* = 0.46) but nevertheless larger than the meta-analysis and in the original study [14]. Our finding that children in the EG did not show the increase in externalising symptoms that the CG did contrasts with Compas et al. [14], who found effects of the intervention on externalising symptoms emerged only at 12-months post-randomisation.

The lack of intervention effects on depressive symptoms (DIKJ) was unexpected and contrasts with the finding that according to the anxiety/depression sub-scale of the YSR, children in the EG showed a greater improvement of symptoms compared to the CG. The lack of effects on the DIKJ may be due to its poor psychometric properties^11^. In the current sample, its reliability was questionable. In comparison, the reliability of the anxiety/depression scale of the YSR was good. Note that DIKJ scores did not correlate with the anxiety/depression sub-scale of the YSR at T2. An alternative explanation for the lack of effects on the DIKJ is that intervention effects only emerge later on in time. Indeed, Compas et al. [14] found that intervention effects on depressive symptoms occurred only 12-months after baseline. Although some trials of preventive interventions for the children of depressed parents have found effects on depressive symptoms immediately after the intervention [22, 48] the majority have not [9, 11, 13, 14, 49]. One explanation for these delayed effects is that children have to be confronted with a certain amount of stress in order to be able to apply their coping skills.

We did not find significant effects of the intervention on parent-reports of children’s internalising or externalising symptoms. This may be because self-reports of internalising symptoms tend to show higher reliability than parent-reports [52], especially if the parent is suffering from an acute episode of depression [3]. Parents and children often perceive children’s psychopathological symptoms differently [8] and in the current sample only small to moderate correlations were found between parent- and child-reported psychopathology (internalising symptoms *r* = 0.527*; externalising symptoms *r* = 0.472**;47** and anxiety/depression *r* = 0.09). Since depression is associated with reduced levels of empathy [43] and an increased self-focus [42] parents in the current study may have been less sensitive to changes in their children’s emotions and behavior [4, 41, 44]. The finding that intervention effects were stronger according to child versus parent reports also replicates the result of the original trial [14] as well as findings of other prevention trials [9].

### Potential mechanisms for the intervention

We found no positive effects of the intervention on any of the hypothesized mechanisms. For example, we found no effects of the intervention on children’s ER whereas the original trial did [12]. One possible explanation for these findings is the different measures used between the two trials (the original trial used the Responses to Stress Questionnaire; RSQ). However, since our measure of ER (FEEL-KJ) included the ER strategies taught in the intervention (acceptance, distraction, positive thinking, positive activities) it is still surprising we did not find any positive effects. It is possible that the expectation of a change of cognitive and behavioral factors over a relatively short period of time is overly optimistic, and will more realistically occur in subsequent follow-up assessments.

In contrast with Compas et al. [14] and our hypotheses, we also found no evidence that the intervention modified parenting style. These null-findings may also reflect measurement issues, since we assessed parenting style by children’s reports whereas in the original trial [12] observations of parent–child interactions were used, which may have been more accurate.

Similarly, to most trials in this field [6, 22, 48] children in the EG showed improved knowledge of depression. However, in contrast to earlier studies, we also found children in the CG showed significantly improved knowledge of depression. This may be because the CG also received a 2–3 h diagnostic session at baseline, as well as birthday and Christmas cards and reminders to complete questionnaires (to maximize data completion). Improvements in children’s knowledge of depression might also be due to the fact that families sought out extra psycho-educative support after being allocated to the CG. In addition, the process of enrolling their family in the intervention may have improved parents’ communication and interaction with their children such that they were able to open up about their mental illness. Feeling more informed about their parents’ illness might have increased feelings of control and security, which may explain in part the internalising symptom reduction [37]. Indeed, similar findings have been observed in other trials of preventive interventions for the children of depressed parents [48, 55].

### The moderating role of parental depression severity

In contrast with positive findings from other preventive trials for the children of depressed parents [6, 7, 22] we did not find any evidence that parent’s depression status (whether they were in an episode of depression at enrollment or not) moderated children’s response to the intervention. It is possible that our failure to find moderating effects was due to the relatively short follow-up time (6 months). However, the original trial found no evidence that parental depression severity moderated children’s outcomes at any of the points in time (2–24 months) [11, 14]. The original trial did not distinguish between parents with a current episode of depression and those without. Meta-analyses with large numbers of RCTs are needed to address the moderating role of parental depression status and/or severity.

Finally, the finding that parents in both groups showed improved depressive symptoms over time contrasts with findings from the original study, in which only those in the EG improved [11, 14]. This supports the notion that families in the CG sought out their own support.

### Acceptance of the intervention

The acceptance of the intervention was rated as good by the participants. The content appeared to be adequate and families felt understood and appreciated. These findings may explain the fact that none of the families in the EG dropped out during the intervention. This finding is crucial and especially relevant for clinical practice and the potential implementation of prevention. Note that 12 families dropped out between randomisation to the EG and the start of the intervention, suggesting some aspects of the intervention may have put families off their initial intentions to participate. Unfortunately, we do not have any data on individual drop-out reasons.

### Strengths

A major strength of the study was the use of standardized clinical interviews (rather than self-reported questionnaires) to characterize parents and children. This increases the validity of the findings and was also intended to maximize ES (the inclusion of parents, who had sub-threshold levels of depression, or of children with elevated symptoms of depression, may have reduced the effects). It is noteworthy that the questionnaire-based outcome variables at the 6-month follow-up are only proxy for depression prevention: the extent to which the intervention has a preventative effect can only be detected by analyzing data on the onset of depression at 15 months (data analysis ongoing). We also sought to include both parent and child measures, although it should be acknowledged that all measures were subjective reports, which are therefore open to bias.

Another strength is the high level of treatment fidelity achieved, which likely reflects the highly manualized intervention, regular supervision and high levels of attendance (which were comparable to the original study; [14]). High treatment fidelity allows more valid attribution of positive findings to the intervention itself.

A final strength of the study is that it represents the first replication of the FGCB intervention, which was originally developed in the U.S. and has not been replicated outside of the research group. Many psychological interventions are evaluated by those who developed them, who are naturally biased to demonstrate the efficacy of their intervention. The findings in the current study suggest the positive findings found by Compas et al. [14] are unlikely to have been influenced by researcher bias.

### Limitations

An important limitation is the relatively high level of missing data (up to 51% in the CBCL at T2), which we had not anticipated. Although numerous measures were taken to obtain data at the three points in time, families reported extremely high levels of strain which hindered their continuing participation. The missing data limits the statistical power of the study, since the sample size calculation (n = 92), which was based on the primary outcome of onset of depression at 15-months, assumed an ES of *d* = 0.6 (power = 0.80, alpha = 0.05). Since participants who completed the questionnaires were probably under less strain than those who did not, the data may be biased. It seems plausible that families under more strain may have benefited more from the intervention, which would suggest our data underestimate the true effect. It should be noted that it is hard to tell how our amount of missing data compares with other studies, since many do not report this quantity [14].

One (uncontrollable) limitation to the external validity of the current study is the poor representativeness of the sample in terms of high socio-economic status, high levels of motivation and a lack of cultural diversity. Families with low socio-economic background, who are underrepresented in this study, are often more affected by depression and may face more stressors (e.g. financial problems, unemployment), and it is unclear if they would benefit equally from the intervention. The reason for our biased sample might be that families from low socio-economic backgrounds have less time to participate in a time-consuming intervention due to work commitments or household tasks. Our sample of parents may also have been more motivated and prepared to open up about their depression than the average family experiencing depression. Reasons families reported for not wanting to participate included fears and shame about opening up about their diagnosis, or overburdening their children with difficult topics. These issues were also raised by parents in a similar preventive intervention when they explained in interviews their reluctance to participate [46]. Parents may often try to hide their illness due to fears about a possible loss of custody [29]. Families that participated were mostly German and, as such, the findings are also less representative for families from ethnic minorities. The importance of “scientific equity” across cultures and within societies when it comes to youth prevention is emphasised by Perrino et al. [45].

Another aspect which limits the interpretation of the findings is the relatively wide age range of the children included. We set the inclusion criterion at 8–17 years in order to maximise chances of recruiting the necessary sample size in the project time period, and because children younger than 8 would not have the cognitive and emotional skills to take part in the intervention. Although this heterogeneity of the children means that they differed in how recently they had been exposed to their parents’ depression, we did at least require the depression to have occurred within the child’s lifetime. Furthermore, the majority of parents had experienced multiple episodes of depression during the child’s lifetime (a single post-natal episode was uncommon). Although the inclusion of children above the age of typical onset of depression (15 years) may have led us to over-sample for particularly resilient children, in reality, the average age of participants was 12 years and the majority (x%) were aged 9–15 years.

Finally, the internal validity of the study findings is also limited, because we did not include an active CG. We included measures of possible mechanisms but an active CG would be necessary to know whether the findings might instead be attributed to “common factors” (e.g. the group setting) [31]. We decided to include a no-intervention CG because our primary aim was to evaluate the benefit of a preventive intervention compared to existing care. Indeed, with this study design, we were able to show how rapidly some outcome variables (e.g. self-reported externalising symptoms) worsened in the CG within just 6 months. This supports longitudinal studies of negative outcomes in children of parents with depression [66].

### Future research

In order to reduce the bias towards a particularly motivated sample with a high socioeconomic status (SES), future studies of prevention interventions may need to consider how they can reduce the stigma and financial costs associated with preventive interventions. Digital interventions administered in a stepped-care approach could be a bridge until families have the courage to participate in face-to-face interventions. Digital interventions may also be more attractive for families who live more remotely. Future research could minimize the strain of data collected on participants by using digital platforms. Future studies could benefit from the inclusion of more objective assessments measures, for example: observations of parent–child interaction [12], physiological measures of stress reactivity (cortisol, electro dermal activity, heart rate) and ecological momentary assessment (EMA). Alternative therapeutic approaches, such as interpersonal therapy (IPT) which have shown to be effective in the treatment [69] and universal prevention [68] of youth depression, may also show promise in the selective prevention of depression in the children of depressed parents. In general, more research and replications of existing trials (outside the U.S.) is needed to achieve more homogenous findings of how preventive interventions should be designed and implemented.

### Clinical implications

The study findings have numerous implications for clinical practice and research. Since the intervention is acceptable to affected families in Germany and the study replicates the positive effects of previous evaluations [11], clinicians or social services working with parents affected by depression may consider using the intervention to support at-risk children. The effect sizes associated with the intervention are relatively high compared to other prevention programs [30]. Thus, although the intervention might be more time consuming and costly than others, it may be a worthwhile investment if the burden of depression is to be reduced. Since parental depression severity does not appear to affect the efficacy of the intervention, clinicians working with currently depressed parents should not be discouraged from supporting their patients in participating in such preventive interventions for their children.

Families who took part in the diagnostic sessions but did not receive the intervention showed reduced depressive symptoms (parents) and improved knowledge of depression (children) over time. This suggests that clinicians working with depressed parents are likely to have a positive effect on families just by starting a conversation about the impacts of parental depression on child outcomes. Nevertheless, clinicians should be aware that whilst the intervention appears to be effective in this sample, it may need to be tailored to appeal to and reach families from less privileged or more diverse backgrounds.

## Conclusion

This is the first study to evaluate the effects of the FGCB preventive intervention for children of parents with depression outside of the original research group [11, 14]. Immediately after the intervention, we found medium to large intervention effects for children’s self-reported internalising, externalising and anxiety-depressive symptoms but no effect on parent-reported psychopathology. There was no evidence that the intervention effects were moderated by whether or not parents were in an episode of depression at baseline. In contrast to our expectations, both groups showed increased knowledge of depression and decreased parental depression over time. Furthermore, we did not find effects of the intervention on potential mechanisms. Future evaluations of preventive interventions should consider ways of designing interventions which appeal to a more representative sample of children of parents suffering from depression.

## Supplementary Information

**Additional file 1**: Overview of prevention intervention sessions.

**Additional file 2**: Effect of intervention on outcomes.

**Additional file 3**: Acceptance ratings (participant feedback for individual sessions).

**Additional file 4**: Acceptance ratings Families’ratings of intervention (individual sessions).

**Additional file 5**: Missing values.

**Additional file 6**: CONSORT statement checklist.

## Data Availability

All data and materials can be provided upon request by the last author (Belinda Platt). We have no conflict of interest. Data transparency statement: We are happy to share an anonymized data set upon request.

## Abbreviations

FGCB: Family-group-cognitive-behavioral prevention program
GuG-Auf: Gesund und Glücklich aufwachsen-translated and adapted FCGB to Germany
EG: Experimental group
CG: Control group
ES: Effect size
ER: Emotion regulation
DIKJ: Depressionsinventar für Kinder und Jugendliche, self-reported symptoms of depression
YSR: Youth self-report form; self-reported psychopathology symptoms
CBCL: Child behavior checklist, parent-reported child psychopathology symptoms
ESI: Erziehungsstil-inventar, parenting style inventory
ASF: Attributionsstil-Fragebogen für Kinder und Jugendliche, child attributional style
FEEL-KJ: Fragebogen zur Erhebung der Emotionsregulation bei Kindern und Jugendlichen, emotion regulation in children
BDI-II: Beck’s depression inventory, depressive symptoms parents

## Footnotes

1 Occasionally varying between 8 and 12 families.

2 Since data analysis is ongoing for T3 and T4 these data are not reported here.

3 SES data were checked by the Winkler Index [56].

4 Data refers to primary parent participating in the study.

5 One family was excluded because the parent had clinically-significant values on the SKID-II.

6 5 parent interviews were not successfully recorded.

7 Since the six subscales of the ASF-KJ are each composed of only 8 items, they may indirectly affect the power of the analysis.

8 in German: Akzeptanz, Ablenkung, positives Denken, positive Aktivitäten.

9 Overly positive statements were perceived by German members of the team as being unauthentic, and whilst group leaders were encouraged to be supportive of families, they often refrained from using the personal pronoun “we” to highlight families’ own responsibility for their actions.

10 The planned analyses reported in the study protocol involved linear regression models for child self-reported (YSR) and parent-reported (CBCL) internalising, externalising and anxiety/depression symptoms as well as children’s self-reported depressive symptoms (DIKJ) [47].

## References

[1] Allgaier A-K, Schiller Y, Schulte-Körne G (2011). Wissens- und Einstellungsänderungen zu Depression im Jugendalter. Entwicklung Und Evaluation Einer Aufklärungsbroschüre.

[2] Allison PD. Handling missing data by maximum likelihood. SAS Global Forum 2012 Statistics and Data Analysis. 2012;1–21.

[3] Angold A, Weissman MM, John K, Merikancas KR, Prusoff BA, Wickramaratne P (1987). Parent and child reports of depressive symptoms in children at low and high risk of depression. J Child Psychol Psychiatry.

[4] Angold A (1995). The development of a short questionnaire for use in epidemiological studies of depression in children and adolescents. Int J Methods Psychiatr Res.

[5] Beardslee WR, Brent DA, Weersing VR, Clarke GN, Porta G, Hollon SD (2013). Prevention of depression in at-risk adolescents: longer-term effects. JAMA Psychiat.

[6] Beardslee WR, Versage EM, Wright EJ, Salt P, Rothberg PC, Drezner K, Gladstone TR (1997). Examination of preventive interventions for families with depression: evidence of change. Dev Psychopathol.

[7] Brent DA (2015). Effect of a cognitive-behavioral prevention program on depression 6 years after implementation among at-risk adolescents: a randomized clinical trial. JAMA Psychiat.

[8] Choudhury MS, Pimentel SS, Kendall PC (2003). Childhood anxiety disorders: parent-child (dis)agreement using a structured interview for the DSM-IV. J Am Acad Child Adolesc Psychiatry.

[9] Clarke GN, Hornbrook M, Lynch F, Polen M, Gale J, Beardslee WR, Seeley J. A randomized trial of a group cognitive intervention for preventing depression in adolescent offspring of depressed parents. Arch Gen Psychiatry. 2001;58(12):1127–1134. 10.1001/archpsyc.58.12.1127. 10.1001/archpsyc.58.12.1127 11735841

[10] Claus N, Marzano L, Löchner J, Starman K, Voggt A, Loy F, Platt B. Qualitative evaluation of a preventive intervention for the offspring of parents with a history of depression. BMC Psychiatry. 2019 10.1186/s12888-019-2273-6 PMC6751651 31533676

[11] Compas BE, Forehand R, Keller G, Champion JE, Rakow A, Reeslund KL (2009). Randomized controlled trial of a family cognitive-behavioral preventive intervention for children of depressed parents. J Consult Clin Psychol.

[12] Compas BE (2010). Coping and parenting: mediators of 12-month outcomes of a family group cognitive-behavioral preventive intervention with families of depressed parents. J Consult Clin Psychol.

[13] Compas BE, Forehand R, Thigpen JC, Keller G, Hardcastle EJ, Cole DA (2011). Family group cognitive-behavioral preventive intervention for families of depressed parents: 18- and 24-month outcomes. J Consult Clin Psychol.

[14] Compas BE, Forehand R, Thigpen J, Hardcastle E, Garai E, McKee L (2015). Efficacy and moderators of a family group cognitive-behavioral preventive intervention for children of parents with depression. J Consult Clin Psychol.

[15] Cracco E, Goossens L, Braet C (2017). Emotion regulation across childhood and adolescence: evidence for a maladaptive shift in adolescence. Eur Child Adolesc Psychiatry.

[16] Cummings EM, Davies PT (1994). Maternal depression and child development. J Child Psychol Psychiatry.

[17] Dearing KF, Gotlib IH (2009). Interpretation of ambiguous information in girls at risk for depression. J Abnorm Child Psychol.

[18] Döpfner M, Berner W, Lehmkuhl G. Handbuch: Fragebogen für Jugendliche. Forschungsergebnisse zur deutschen Fassung der Youth Self-Report Form (YSR) der Child Behavior Checklist. 1994.

[19] Döpfner M, Schmeck K, Berner W. Handbuch: Elternfragebogen über das Verhalten von Kindern und Jugendlichen. Forschungsergebnisse zur deutschen Fassung der Child Behavior Checklist (CBCL/4-18) 1994.

[20] Downey G, Coyne JC (1990). Children of depressed parents: an integrative review. Psychol Bull.

[21] Dunbar JP, McKee L, Rakow A, Watson KH, Forehand R, Compas BE (2013). Coping, negative cognitive style and depressive symptoms in children of depressed parents. Cogn Ther Res.

[22] Garber J, Clarke GN, Weersing VR, Beardslee WR, Brent DA, Gladstone TRG (2009). Prevention of depression in at-risk adolescents: a randomized controlled trial. NIH Public Access.

[23] Goodman S, Gotlib I (1999). Risk for psychopathology in the children of depressed mothers: a developmental model for understanding mechanisms of transmission. Psychol Rev.

[24] Goodman SH, Rouse MH, Connell AM, Broth MR, Hall CM, Heyward D (2011). Maternal depression and child psychopathology: a meta-analytic review. Clinical Child an.

[25] Grob A, Smolenski C (2005). Fragebogen Zur Erhebung Der Emotionsregulation Bei Kindern Und Jugendlichen (FEEL-KJ). [Questionnaire to assess children and adolescents’ emotion regulation].

[26] Hart CH, Newell L, Olsen S. Parenting skills and social-communicative competence in childhood. In: Handbook of communication and social interaction skill. 2003:753–797.

[27] Hautzinger M, Bailer M, Worall H, Keller F. Beck-Depressions-Inventar (BDI). German version. Test manual.(Bearbeitung der deutschen Ausgabe. Testhandbuch.). Göttingen: Huber. 1994.

[28] Hayden EP, Hankin BL, Mackrell SV, Sheikh HI, Jordan PL, Dozois DJ (2014). Parental depression and child cognitive vulnerability predict children’s cortisol reactivity. Dev Psychopathol.

[29] Hearle J (1999). A survey of contact with offspring and assistance with child care among parents with psychotic disorders. Psychiatr Serv.

[30] Hetrick SE (2016). Cognitive Behavioural Therapy (CBT), Third-Wave CBT and Interpersonal Therapy (IPT) based interventions for preventing depression in children and adolescents. Cochrane Database Syst Rev.

[31] Huibers MJH (2014). Predicting response to cognitive therapy and interpersonal therapy, with or without antidepressant medication, for major depression: a pragmatic trial in routine practice. J Affect Disord.

[32] Keller GA, Gottlieb DT (2012). Reducing major depression in children at high risk: opportunities for prevention. Int J Psychiatry Med.

[33] Kirmayer LJ (2007). Psychotherapy and the cultural concept of the person. Transcult Psychiatry.

[34] Kovacs M. The Children’s Depression, Inventory (CDI). North Towanda, NY: Multi-Health System. 1992. 10.3724/SP.J.1041.2015.01004.

[35] Krohne HW, Pulsack A. Erziehungsstil-Inventar. Goettingen: Beltz Test GmbH. 1991.

[36] Kühner C. Fragebogen zur Depressionsdiagnostik nach DSM-IV. 1997.

[37] Lenz A. Vorstellungen der Kinder über die psychische Erkrankung ihrer Eltern. Eine explorative Studie. Praxis Der Kinderpsychologie Und Kinderpsychiatrie. 2005;54(5):382–398. 16032948

[38] Loechner J, Starman K, Galuschka K, Tamm J, Schulte-Körne G, Rubel J, Platt B. Preventing depression in the offspring of parents with depression: a systematic review and meta-analysis of randomized controlled trials. Clin Psychol Rev. 2018:1–14. 10.1016/j.cpr.2017.11.009. 10.1016/j.cpr.2017.11.009 29305152

[39] Lovejoy MC, Graczyk PA, O’Hare E, Neuman G (2000). Maternal depression and parenting behavior: a meta-analytic review. Clin Psychol Rev.

[40] Mason WA, Haggerty KP, Fleming AP, Casey-Goldstein M (2012). Family intervention to prevent depression and substance use among adolescents of depressed parents. J Child Fam Stud.

[41] Messer S, Angold A, Costello J, Loeber R (1995). Development of a short questionnaire for use in epidemiological studies of depression in children and adolescents: factor composition and structure across development. Int J Methods Psychiatr Res.

[42] Morris SJ, Kanfer FH (1983). Altruism and depression. Pers Soc Psychol Bull.

[43] O’Connor LE, Berry JW, Weiss J, Gilbert P (2002). Guilt, fear, submission, and empathy in depression. J Affect Disord.

[44] Orchard F, Pass L, Marshall T, Reynolds S (2017). Clinical characteristics of adolescents referred for treatment of depressive disorders. Child Adolesc Mental Health.

[45] Perrino T (2015). Toward scientific equity for the prevention of depression and depressive symptoms in vulnerable youth. Prev Sci.

[46] Phikala H, Johansson EE (2008). Longing and fearing for dialogue with children: depressed parents’ way into Beardslee’s preventive family intervention. Nord J Psychiatry.

[47] Platt B, Pietsch K, Krick K, Oort F, Schulte-Körne G (2014). Study protocol for a randomised controlled trial of a cognitive-behavioural prevention programme for the children of parents with depression: The PRODO trial. BMC Psychiatry.

[48] Punamäki RL, Paavonen J, Toikka S, Solantaus T (2013). Effectiveness of preventive family intervention in improving cognitive attributions among children of depressed parents: a randomized study. J Family Psychol.

[49] Sanford M, Byrne C, Williams S, Atley S, Ridley T, Miller J, Allin H (2003). A pilot study of a parent-education group for families affected by depression. Can J Psychiatry.

[50] Schäfer JÖ, Naumann E, Holmes EA, Tuschen-Caffier B, Samson AC (2016). Emotion regulation strategies in depressive and anxiety symptoms in youth: a meta-analytic review. J Youth Adolesc.

[51] Schneider S, Margraf J (2011). Diagnostic interview for psychiatric disorders [Diagnostisches Interview bei psychischen Störungen].

[52] Schneider S, In-Albon T, Nuendel B, Margraf J (2013). Parental panic treatment reduces children’s long-term psychopathology: a prospective longitudinal study. Psychother Psychosom.

[53] Sfärlea A, Löchner J, Neumüller J, Salemink E, Asperud Thomsen L, Starman K (2019). Passing on the half-empty glass: a transgenerational study of interpretation biases in children at risk for depression and their parents with depression. J Abnorm Psychol.

[54] Silk JS, Shaw DS, Forbes EE, Lane TL, Kovacs M (2006). Maternal depression and child internalizing: the moderating role of child emotion regulation. J Clin Child Adolesc Psychol.

[55] Solantaus T, Juulia Paavonen E, Toikka S, Punamäki RL (2010). Preventive Interventions in Families with Parental Depression: Children’s Psychosocial Symptoms and Prosocial Behaviour. Eur Child Adolesc Psychiatry.

[56] Starman, K. Baseline differences and intervention effects of the “Gesund und Glücklich Aufwachsen (GUG-Auf)” prevention program for children of depressed parents. Retrieved from https://edoc.ub.uni-muenchen.de/22238/. 2018.

[57] Stephens M, Smith NJ, Donnelly P (2001). A new statistical method for haplotype reconstruction from population data. Am J Hum Genet.

[58] Stieglitz R-D (2002). Familientherapie aus verhaltenstherapeutischer Sicht. Paar- und Familientherapie.

[59] Stiensmeier-Pelster J, Schürmann M, Eckert C, Pelster A. Attributionsstil-Fragebogen für Kinder und Jugendliche (ASF-KJ). Handanweisung. Göttingen: Hogrefe. 1994.

[60] Stiensmeier-Pelster J, Braune-Krickau M, Schürmann M, Duda K. *DIKJ.* Depressionsinventar für Kinder und Jugendliche. Göttingen: Hogrefe. 2014.

[61] Suppiger A, In-Albon T, Herren C, Bader K, Schneider S, Margraf J (2008). Reliabilität des Diagnostischen Interviews bei Psychischen Störungen (DIPS für DSM-IV-TR) unter klinischen Routinebedingungen. Verhaltenstherapie.

[62] Tronick E, Reck C (2009). Infants of depressed mothers. Harv Rev Psychiatry.

[63] Unnewehr S, Schneider S, Margraf J, Unnewehr S, Margraf J, Schneider S, Margraf J (2009). Kinder-DIPS-Diagnostisches Interview bei psychischen Störungen im Kindes- und Jugendalter. 2. aktualisiterte und.

[64] Weiß RH. *CFT**20-R.**Grundintelligenztest**Skala**2.**Revision.* . Göttingen: Hogrefe Verlag GmbH & Co. KG. 2006

[65] Weiß RH (2006). Grundintelligenztest Skala 2-Revision-(CFT 20-R).

[66] Weissman M, Warner V, Wickramaratne P, Moreau D, Olfson M. Offspring of depressed parents. 10 Years later. Arch Gen Psychiatry. 1997;54(10):932–40. 10.1001/archpsyc.1997.01830220054009 9337774

[67] Weissman MM, Wickramaratne P, Nomura Y, Warner V, Verdeli H, Pilowsky DJ (2005). Families at high and low risk for depression: a 3-generation study. Arch Gen Psychiatry.

[68] Young CJ, Davidson JA, Gross AM (2010). Handbook of clinical psychology competencies.

[69] Zhou X (2015). Comparative efficacy and acceptability of psychotherapies for depression in children and adolescents: a systematic review and network meta-analysis. World Psychiatry.

[70] Zietlow AL, Schlüter MK, Nonnenmacher N, Müller M, Reck C (2014). Maternal self-confidence postpartum and at pre-school age: the role of depression, anxiety disorders, maternal attachment insecurity. Matern Child Health J.

