# Supplementary material for: A randomised controlled trial of a family-group cognitive-behavioural (FGCB) preventive intervention for the children of parents with depression: short-term effects on symptoms and possible mechanisms
Source: Child Adolesc Psychiatry Ment Health. 2021 Oct 1;15:54. doi: 10.1186/s13034-021-00394-2 (PMC8487152; doi:10.1186/s13034-021-00394-2)
Supplement: Supplementary file 1 — Additional file 1: Overview of prevention intervention sessions. [file 13034_2021_394_MOESM1_ESM.docx]

***Supplement 1: Overview of prevention intervention sessions***

|  | | |
| --- | --- | --- |
| Session | **Family** | |
| 1. | Introduction, causes and symptoms of depression | |
| 2. | Reactions to stress | |
|  | Introducing coping strategies | |
|  | **Children** | **Parents** |
| 4. | Acceptance | Positive parenting & attention |
| 5. | Positive activities | Ignoring and positive parenting |
| 6. | Positive thinking | Family rules, giving instructions |
| 7. | Distraction | Monitoring, token economy |
| 8. | Role plays | Positive parenting and depression, supporting children with coping |
| 9-12 | Booster-sessions | |

*Note*: all sessions start and end in the family- and group- setting with 3-5 families á 120 min., sessions 4-8 are partly separated
