# Supplementary material for: A randomised controlled trial of a family-group cognitive-behavioural (FGCB) preventive intervention for the children of parents with depression: short-term effects on symptoms and possible mechanisms
Source: Child Adolesc Psychiatry Ment Health. 2021 Oct 1;15:54. doi: 10.1186/s13034-021-00394-2 (PMC8487152; doi:10.1186/s13034-021-00394-2)
Supplement: Supplementary file 2 — Additional file 2: Effect of intervention on outcomes. [file 13034_2021_394_MOESM2_ESM.docx]

***Supplement 2 Effect of intervention on outcomes***

 
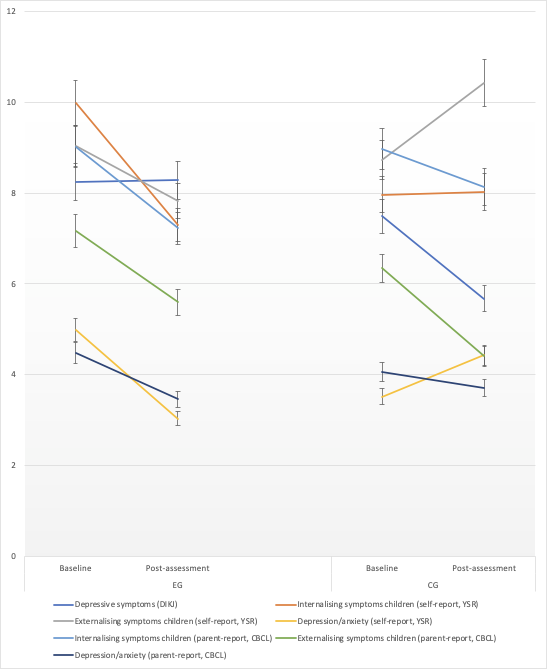


*Note*. All values are raw values. anx./depr. = anxiety/depressive symptoms; CBCL = Child Behaviour Checklist; DIKJ = Depressions-Inventar für Kinder und Jugendliche; ext. = externalising symptoms, int. = internalising symptoms, YSR = Youth Self-Report, error indicators are 5% CI.
