# Supplementary material for: A randomised controlled trial of a family-group cognitive-behavioural (FGCB) preventive intervention for the children of parents with depression: short-term effects on symptoms and possible mechanisms
Source: Child Adolesc Psychiatry Ment Health. 2021 Oct 1;15:54. doi: 10.1186/s13034-021-00394-2 (PMC8487152; doi:10.1186/s13034-021-00394-2)
Supplement: Supplementary file 3 — Additional file 3: Acceptance ratings (participant feedback for individual sessions). [file 13034_2021_394_MOESM3_ESM.docx]

***Supplement 3 Families’ ratings of intervention (average across all sessions)***

Feedback Questionnaire

GuG auf – Gesund und Glücklich Aufwachsen! Session # Date:

Please answer a few questions about tonight’s session to help us improve these sessions. Please circle the answer that best describes how you are feeling.

1. How well did you understand the material that was covered in tonight’s session?

1 2 3 4 5

not at all somewhat very much

2. How much did you participate in session activities and discussions tonight?

1 2 3 4 5

none some a lot

3. How comfortable did you feel participating in tonight’s session?

1 2 3 4 5

not at all somewhat very much

4. How much did you feel understood and supported by the group leaders tonight?

1 2 3 4 5

not at all somewhat very much so

5. After discussing last week’s homework in tonight’s session, how well do you feel you understand the homework?

1 2 3 4 5

not at all somewhat very much

6. How helpful did you find the activities in tonight’s session?

1 2 3 4 5

not at all somewhat very much

Please write in this space below anything else that you’d like to let us know about tonight’s session. Thank you!

Results across all families and sessions

| **Parents** | M | SD | Min | Max |
| --- | --- | --- | --- | --- |
| Understanding the content | 4.64 | 0.62 | 1 | 5 |
| Active participation | 4.02 | 0.75 | 2 | 5 |
| Feeling comfortable | 4.27 | 0.78 | 1 | 5 |
| Feeling understood / supported | 4.59 | 0.73 | 2 | 5 |
| Understanding the exercises | 4.44 | 0.65 | 1 | 5 |
| Usefulness of exercises | 4.14 | 0.77 | 1 | 5 |
| **Children** |  |  |  |  |
| Understanding the content | 4.50 | 0.71 | 1 | 5 |
| Active participation | 4.01 | 1.07 | 1 | 5 |
| Feeling comfortable | 4.48 | 0.81 | 1 | 5 |
| Feeling understood / supported | 4.52 | 0.71 | 1 | 5 |
| Understanding the exercises | 4.38 | 0.89 | 1 | 5 |
| Usefulness of exercises | 4.33 | 0.82 | 1 | 5 |

*Note .*Min = minimum; Max = maximum.1 = lowest rating; 5 = highest rating.
