# Supplementary material for: A randomised controlled trial of a family-group cognitive-behavioural (FGCB) preventive intervention for the children of parents with depression: short-term effects on symptoms and possible mechanisms
Source: Child Adolesc Psychiatry Ment Health. 2021 Oct 1;15:54. doi: 10.1186/s13034-021-00394-2 (PMC8487152; doi:10.1186/s13034-021-00394-2)
Supplement: Supplementary file 4 — Additional file 4: Acceptance ratings Families’ratings of intervention (individual sessions). [file 13034_2021_394_MOESM4_ESM.docx]

***Supplement 4 Acceptance ratings Families’ratings of intervention (individual sessions)***


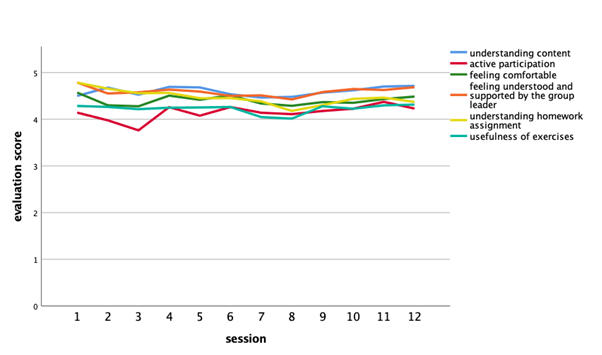


*Note*. N=135 children of n=100 families, signif. codes: 0 ‘***’ 0.001 ‘**’ 0.01 ‘*’ 0.05 ‘.’ 0.1 ‘ ’
