# Supplementary material for: A randomised controlled trial of a family-group cognitive-behavioural (FGCB) preventive intervention for the children of parents with depression: short-term effects on symptoms and possible mechanisms
Source: Child Adolesc Psychiatry Ment Health. 2021 Oct 1;15:54. doi: 10.1186/s13034-021-00394-2 (PMC8487152; doi:10.1186/s13034-021-00394-2)
Supplement: Supplementary file 5 — Additional file 5: Missing values. [file 13034_2021_394_MOESM5_ESM.docx]

***Supplement 5: Missing values***

Percentage of missing values at baseline and post-assessment

|  | BDI-II | DIKJ | CBCL | YSR | FEEL-KJ | ASF | Knowledge of Depression | ESI |
| --- | --- | --- | --- | --- | --- | --- | --- | --- |
| Baseline | 10 | 29 | 25 | 25 | 19 | 26 | 26 | 24 |
| Post-assessment | 50 | 58 | 50 | 48 | 47 | 46 | 48 | 48 |

*Note.* BDI-II = Beck’s Depression Inventory; DIKJ = Depressions-Inventar für Kinder und Jugendliche; CBCL = Child Behaviour Checklist; YSR = Youth Self-Report; FEEL-KJ = Fragebogen zur Erhebung der Emotionsregulation bei Kindern und Jugendlichen; ASF = Attributionsstil-Fragebogen für Kinder und Jugendliche; ESI = Erziehungsstil-Inventar.
